# LC-MS/MS Determination of Isoprostanes in Plasma Samples Collected from Mice Exposed to Doxorubicin or Tert-Butyl Hydroperoxide

**DOI:** 10.3390/ijms14036157

**Published:** 2013-03-18

**Authors:** Monika Janicka, Agata Kot-Wasik, Jolanta Paradziej-Łukowicz, Grażyna Sularz-Peszyńska, Agnieszka Bartoszek, Jacek Namieśnik

**Affiliations:** 1 Department of Analytical Chemistry, Chemical Faculty, Gdańsk University of Technology, Narutowicza 11/12, 80-233 Gdańsk, Poland; E-Mails: agata@chem.pg.gda.pl (A.K.-W.); jacek.namiesnik@pg.gda.pl (J.N.); 2 Tri-City Central Animal Laboratory Research and Service Centre of the Medical University of Gdansk, Medical University of Gdansk, 80-210 Gdańsk, Poland; E-Mails: lukowicz@gumed.edu.pl (J.P.-Ł.); gsularz@gumed.edu.pl (G.S.-P.); 3 Department of Food Chemistry, Technology and Biotechnology, Chemical Faculty, Gdańsk University of Technology, Narutowicza 11/12, 80-233 Gdańsk, Poland; E-Mail: agnieszka.bartoszek@pg.gda.pl

**Keywords:** isoprostanes, doxorubicin, plasma biomarkers of oxidative stress, LC-MS/MS

## Abstract

Isoprostanes are stable products of arachidonic acid peroxidation and are regarded as the most reliable markers of oxidative stress *in vivo*. Here we describe the LC-MS/MS procedure enabling simultaneous determination of four regioisomers (8-*iso* prostaglandin F_2α_, 8-*iso*-15(R)-prostaglandin F_2α_, 11β-prostaglandin F_2α_, 15(R)-prostaglandin F_2α_) in plasma samples collected from mice. The four plasma isoprostanes are determined by LC–ESI-MS/MS with deuterated 8-*iso*-PGF_2α_-d_4_ as an internal standard (I.S.). For plasma samples spiked with the isoprostanes at a level of 200 pg/mL each, the method imprecision has been below 7.1% and mean inaccuracy equaled 8.7%. The applicability of the proposed approach has been verified by the assessment of changes in isoprostane levels in plasma samples derived from mice exposed to *tert*-butyl hydroperoxide (TBHP), a model inducer of oxidative stress, or to antitumor drug doxorubicin (DOX) known for potent stimulation of redox cycling. Compared to the control group of mice, both oxidative stress inducers tested increased the levels of three out of four isoprostanes in exposed animals; 11β-prostaglandin F_2α_ being the exception. The greatest rise was observed in the case of 15(R)-prostaglandin F_2α_, by about 50% and 70% in plasma samples derived from mice exposed to DOX and TBHP, respectively.

## 1. Introduction

Isoprostanes are a relatively newly discovered class of compounds formed *in vivo* as a result of non-enzymatic oxidation of polyunsaturated fatty acids. The isoprostanes arising by free radical-catalyzed peroxidation of arachidonic acid (AA), so called F_2_-*iso*P, were the first to be described by Mihelich [1]. Their occurrence has now been established as the most reliable marker of oxidative stress, while the measurement of their concentration in biological samples is the most recommended approach to the assessment of redox status *in vivo*, providing an important tool for exploring the role of oxidative processes in the pathogenesis of human disease [2,3]. The elevated levels of F_2_-*iso*P have been observed in a number of conditions associated with increased abundance of reactive oxygen species such as neurodegenerative diseases (Alzheimer, Huntington or multiple sclerosis) [4–6], alcoholic and non-alcoholic liver diseases [7,8], asthma [9], pulmonary disease [10] or diabetes [11]. However, the usefulness of these products of oxidative stress is particularly emphasized in the case of atherosclerotic patients [12] and suggested to be a valuable risk marker for coronary heart disease [13]. The role of isoprostanes in evaluation of cancer risk and efficacy of chemoprevention has also been postulated [14].

The F_2_*-iso*P are not the only isoprostanes found *in vivo*; other types include F_3_*-iso*P formed from eicosapentaenoic acid (EPA) [15] and the most structurally diverse products of docosahexaenoic acid (DHA) non-enzymatic oxidation [16]. The EPA- and DHA-derived isoprostanes seem to play multiple biological roles, so F_2_*-iso*P remain the major indicators of oxidative stress.

The isoprostanes are commonly measured in plasma or urine by well-established gas chromatography–mass spectrometry (GC-MS) and enzyme-linked immunosorbent assays (ELISA). Progress in this field has been the subject of several review articles [12–18]. Even though the GC-MS method is sensitive, the procedure is usually tedious and time-consuming due to the fact that a derivatization step is required prior to separation [19]. Typically, isoprostanes are converted to pentafluorobenzyl esters by treatment with pentafluorobenzyl bromide [20,21]. The main drawback of commercially available ELISA is that a cross-linking reaction can be observed and each assay kit can only measure just one isomer of F2-isoprostanes [22]. However, when sensitivity and selectivity issues in the analysis of biomolecules arise, among various chromatographic methods, liquid chromatography-tandem mass spectrometry (LC-MS/MS) is optimal to overcome such problems. LC-MS/MS advantages include relatively short run times and higher sensitivity and selectivity, especially in the case of such samples as e.g., plasma that contain a considerable amount of coeluting interferences. LC-MS/MS method has been proven to be powerful for simultaneous measurement of different isomers of F_2_-isoprostanes [23–25]. Moreover, to achieve accurate quantitation at extremely low concentrations, there is the possibility of using nonradioactive isotope-labeled standards to compensate for the loss of analyte during sample preparation, which has been the most critical step for eliminating the matrix effect for analysis of biomarkers by mass spectrometry.

This research concentrated on AA-derived isoprostanes that are regarded as the most useful markers of oxidative stress *in vitro* and *in vivo*, and most importantly in studies involving humans. It was aimed at establishing a simple and specific HPLC-MS/MS method for simultaneous determination of four regioisomers that are formed upon free radical oxidation of AA. The additional assumption was that the method should be applicable to measurements where only a very limited amount of biological material is accessible. Such a restriction can be expected during experiments involving small animals (e.g., mouse plasma as a sample), but also in lipidomic or metabolomic studies or in clinical settings, where biological samples must suffice for multiple different analytical procedures. The developed chromatographic protocol was then used to examine the relationship between induction of oxidative stress and generation of F_2_*-iso*P by comparing the concentrations of four regioisomers in the plasma of mice treated with doxorubicin (DOX) or *tert*-butyl hydroperoxide (TBHP) exhibiting prooxidant properties and control group of animals.

## 2. Results and Discussion

### 2.1. Development of HPLC-MS/MS Conditions

**Figure 1:**
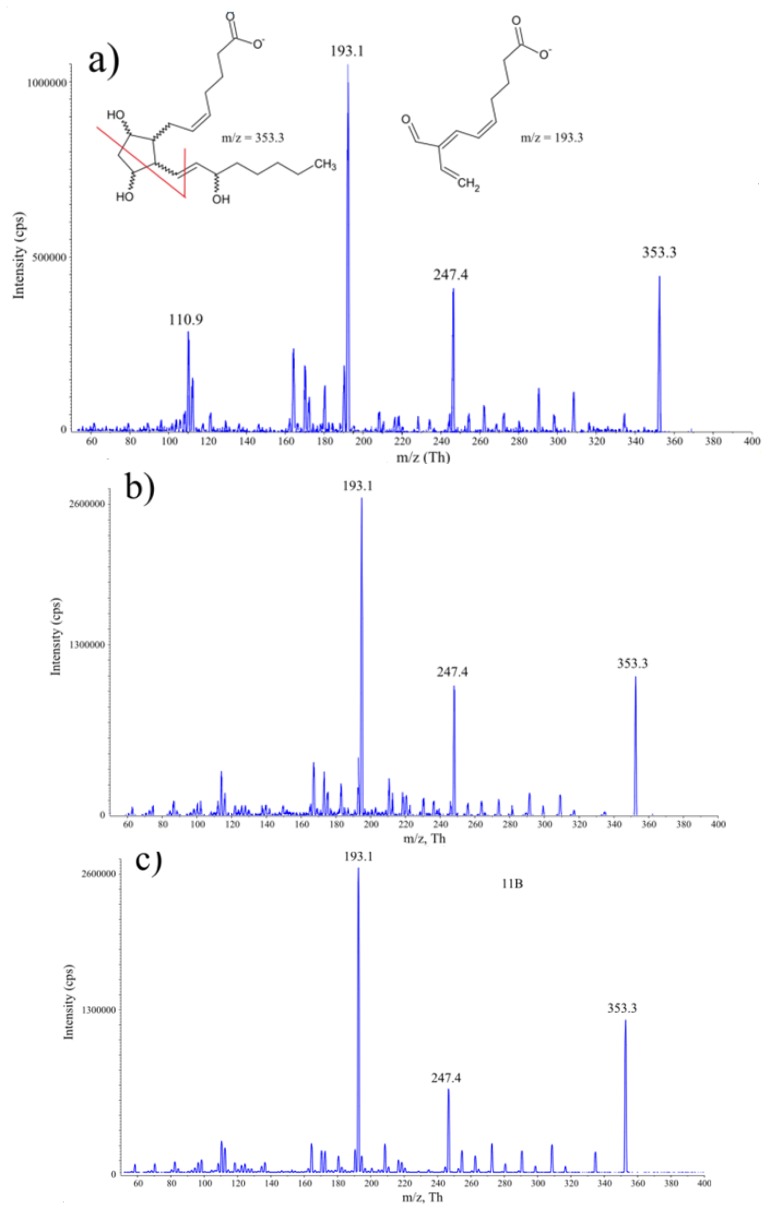

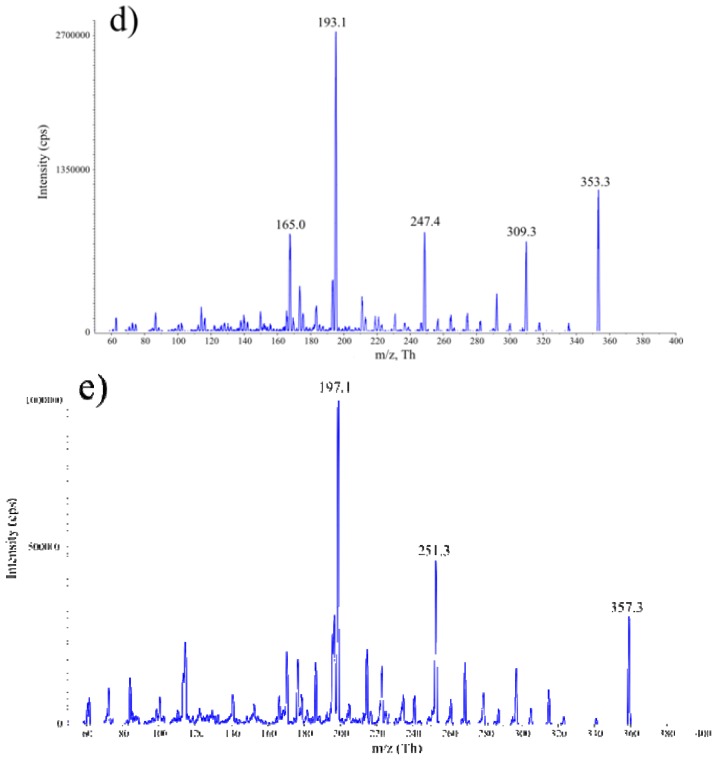
Mass spectrum of (**a**) 8*-iso*P; (**b**) 8,15*-iso*P; (**c**) 11*-iso*P; (**d**) 15*-iso*P product ion *m*/*z* 353.3 and (**e**) IS product ion m/z 357.3. The most intense fragment ion m/z 193.3 for isoprostanes and *m*/*z* 197.1 were selected for quantitation. Chemical structures show proposed fragmentation pattern of isoprostanes.

The parameters of chromatographic resolution were developed with the aid of four isoprostane regioisomers arising upon AA radical-catalysed oxidation: 8*-iso*P, 8,15*-iso*P, 11*-iso*P and 15*-iso*P. The quantitative determination of analytes was optimized based on 8*-iso* prostaglandin F_2α_-d_4_ that served as the internal standard. Deprotonated molecules of all four isoprostanes were detected at *m*/*z* 353 during negative ion electrospray mass spectrometry. The representative product ion tandem mass spectrum of 8*-iso*P is shown in Figure 1. The most intense product is ion m/z 193 and such ion transitions *m*/*z* 353.3→193 are characteristic for all isoprostanes. Consequently, transition to m/z 193 was used for the detection and quantitation of analytes investigated.

**Figure 2:**
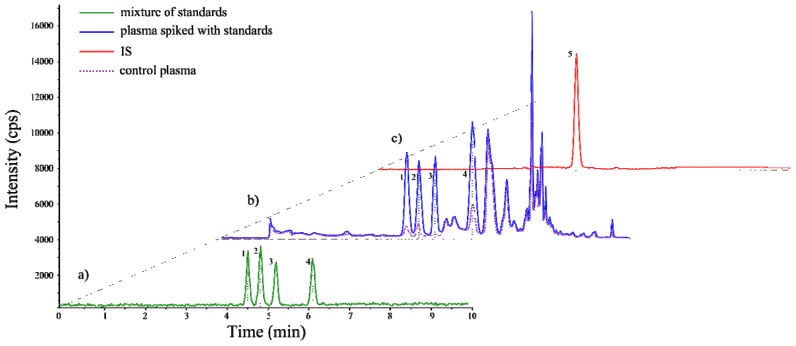
LC-MS/MS chromatogram of (**a**) mixture of four isoprostane standards (1. 8,15*-iso*P; 2. 8*-iso*P; 3. 11*-iso*P; 4. 15*-iso*P); (**b**) control plasma sample and plasma sample spiked with four isoprostanes after SPE sample preparation; (**c**) internal standard (I.S.).

In Figure 2, the LC-MS/MS chromatograms of a mixture of standards 8*-iso*P, 8,15*-iso*P, 11*-iso*P, 15*-iso*P (100 pg/mL each compound), I.S. and exemplary mouse plasma sample spiked with standards are compared. The mouse plasma sample (100 μL) and water sample (100 μL) were spiked with four isoprostane (5 μL of 20 ng/mL), internal standard (10 μL of 250 ng/mL) solutions and submitted to SPE purification procedure described under Materials and Methods. As can be seen, the procedure developed ensured that regioisomers were clearly separated from each other and no additional peaks appeared in the chromatogram obtained for the solution of standards (Figure 2a). In the case of real sample however, the additional peaks of non-identified analytes exhibiting spectral properties of isoprostanes were detected (Figure 2b). Importantly, these unknown compounds did not coelute with the determined ones. This suggests that with the aid of appropriate standards, the method enables also monitoring of other AA-derived isoprostanes, broadening the scope of applicability of the developed procedure.

### 2.2. Method Validation

Calibration curves were prepared using standard addition method typically performed for samples containing already analytes. Twofold quantitation has been done: solutions of isoprostanes together with deuterated standard (applied for the compensation of detector response) were prepared in water as well as in control plasma samples. Both types of matrices were spiked with isoprostane standard solutions at seven different concentrations ranging from 50 to 5000 pg/mL. In both cases samples were prepared according to SPE protocol and extracts were analyzed. The calibration curves were generated for the relationship between the peak area determined for individual substances (8*-iso*P, 8,15*-iso*P, 11*-iso*P, 15*-iso*P) and the peak area of I.S. at the particular analyte concentration. The analysis for each calibration solution was performed in triplicate. Obtained data showed no significant difference between both calibration curves (for water and plasma samples slopes remained the same). Matrix effects were reduced by the application of SPE procedure. Differences in intercepts are caused by the basal level of isoprostans in used plasma samples (Figure 2b). Limits of detection (LODs) and limits of quantitation (LOQs) were determined by serial dilution of spiked water samples. LODs and LOQs were evaluated on the basis of a signal-to-noise ratio of 3 and 10, respectively. The calibration data including calibration line equations, determination of coefficients, limits of detection and quantitation are listed in Table 1. All calibration curves showed good linearity throughout the used range of concentrations with a regression coefficient of 0.999. This confirms the reliability of isoprostane quantitation by the proposed LC-MS/MS procedure.

The trueness, repeatability and reproducibility of the method were tested for samples of mouse plasma fortified with isoprostanes under study. To determine trueness, the plasma samples were spiked with standards at a different concentration level (50, 100, 250 pg/mL). Every sample was analyzed in triplicate. The results obtained by comparing the peak areas of spiked and processed plasma samples with the corresponding standard solutions (matrix free) analyzed without sample preparation are presented in Table 2.

Regardless of the spiking level, satisfactory recoveries ( $\frac{97.9}{100.9}\%$), with relative standard deviations (RSDs) below 9%, were obtained for all isoprostanes tested. The repeatability and reproducibility of the method was assessed by replicate analyses of plasma samples at one spiking level (200 pg/mL). In the first series of experiments, the samples of plasma were analyzed three times a day (nine samples of plasma) to determine the intra-day repeatability expressed as RSD of within-day averages. Then, the analyses were repeated over three consecutive days to calculate inter-day reproducibility expressed as RSD of between-days averages. The results of these studies are presented in Table 3.

The intra-day repeatability was between $\frac{1.9}{7.1}\%$. The inter-day reproducibility was between $\frac{0.2}{2.6}\%$. All these data confirm the satisfactory reproducibility of the method developed.

### 2.3. Real Sample Analysis

**Figure 3:**
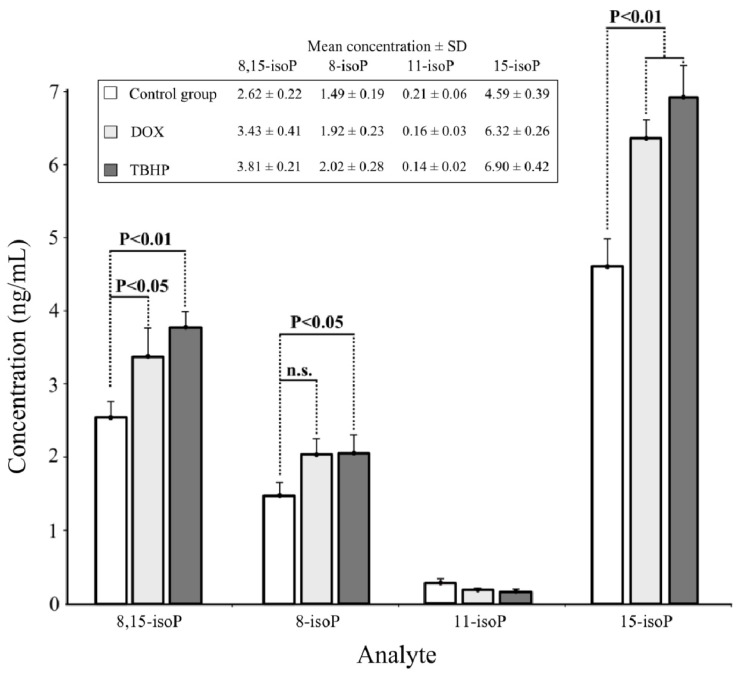
The LC-MS/MS determination of four isoprostanes in plasma of mice exposed to oxidative stress: Control group—non-treated animals, DOX—animals administered *i.p.* 20 mg/kg of DOX, TBHP—animals administered *i.p.* 1.35 mg/kg of TBHP. The results are means ± SD. The statistical significance of differences between plasma samples from control *vs.* exposed animals was assessed by ANOVA with Dunnett’s multiple comparison test (n.s.—not significant).

The applicability of the developed LC-MS/MS method was verified by the determination of AA-derived isoprostanes in plasma samples collected from mice exposed to oxidative stress induced as a result of *i.p.* administration of chemical prooxidants acting via different modes. TBHP as a hydroperoxide is capable of direct oxidation of polyunsaturated fatty acids and is frequently used to stimulate lipid peroxidation *in vivo*[26]. DOX, a well-established antitumor drug, is known to promote cascade of reactive oxygen species as a result of enzymatically catalyzed redox cycling with molecular oxygen [27]. The analysis of plasma samples revealed that exposure of mice to oxidative stress stimulated by DOX and TBHP highly increased the formation of 8,15*-iso*P (by 48% and 66%, respectively) and 15*-iso*P (by 50% and 69%, respectively) compared to plasma samples collected from non-treated animals (Figure 3). Especially, 15*-iso*P seems to be a very sensitive marker of lipid peroxidation *in vivo*. The increase of 8*-iso*P generation in comparison with control samples, reached the statistical significance only in the case of TBHP treatment. In the case of real samples for which concentration of isoprostanes was higher than 5 ng/mL, a step of dilution (1:1) with mobile phase and re-analysis was done. The fourth determined regioisomer—11*-iso*P—appeared to decrease in animals exposed to oxidative stress, however this change was not significant compared to controls. Also t-test revealed no statistically significant differences between TBHP and DOX as stimulants of lipid peroxidation when assessed based on 11-*iso*P content. The most surprising was the observation that 8*-iso*P, the most frequently used and regarded as the most reliable isoprostane biomarker of *in vivo* lipid peroxidation, turned out to give the least convincing evidence of exposure of animals to oxidative stress. All these data taken together support the soundness of the determination of four, representing class VI, AA-derived isoprostanes for the evaluation of organism’s redox status.

## 3. Materials and Methods

### 3.1. Reagents and Materials

HPLC-MS grade methanol and acetonitrile were from J.T.Baker (ŁódŸ, Poland). Formic acid was from P.O.Ch (Gliwice, Poland). Ultrapure water was prepared with an HPLC5 system (Hydrolab, Poland). Doxorubicin (DOX) and *tert*-butyl hydroperoxide (TBHP) were from Sigma-Aldrich Co Ltd. (Warsaw, Poland). Certified standards of isoprostanes including 8-*iso* prostaglandin F_2α_ (8*-iso*-PGF_2α_, **8*-iso*P**), 8-*iso*-15(R)-prostaglandin F_2α_, (8*-iso*-15(R)-PGF_2α_, **8,15-*iso*P**), 11β-prostaglandin F_2α_ (11β-PGF_2α_, **11*-iso*P**), 15(R)-prostaglandin F_2α_ (15(R)-PGF_2α_, **15*-iso*P**) were obtained from SPI-BIO (Montigny le Bretonneux, France) as was the deuterated internal standard 8-*iso* prostaglandin F_2α_ -d_4_ (8*-iso*-PGF_2α −_-d_4_, **I.S.**). The stock solutions (1 mg/mL) of four regioisomers used: 8*-iso*P, 8,15*-iso*P, 11*-iso*P and 15*-iso*P, as well as of internal standard I.S. were prepared in methanol, aliquoted and stored at −80 °C. Just before use, the stock solutions were diluted with methanol:acetonitrile:water (25:25:50 *v*/*v*/*v*) to generate a series of calibration solutions (concentration range 0.01–5 ng/mL).

### 3.2. Exposure of Mice to Oxidative Stress Inducers

The animals, inbred male F1 (C57BL/6xDBA/2) mice, were housed in plastic cages and were given standard granulated feed and tap water *ad libitum*. The animals were randomized and divided into three test groups including 6 animals in control (non-treated) and 5 animals in tested groups. One of the exposed to oxidative stress groups of animals was injected *i.p*. with a model oxidant TBHP in a single dose of 1.35 mg/kg 2 h before the collection of biological material. The other inducer of oxidative stress—doxorubicin (DOX)—was applied *i.p*. as a single toxic dose (20 mg/kg) for 2 h. Controls received an equivalent volume of saline. After treatment period, animals were anesthetized with Isofluranum^®^. All experimental procedures involving animals were approved by the Local Ethical Committee (For Use of Laboratory Animals) located at Medical University of Gdańsk, Poland, and were in conformity with national and international laws (EEC Council Directive 86/609).

### 3.3. Collection of Plasma Samples

Blood (typically 0.5 mL) was collected from hearts and transferred to centrifuge tubes coated with EDTA as an anticoagulant (1.5 mL vial volume, Medlab, Poland) and centrifuged in a way enabling separation of both plasma and white blood cells in a single run (5 min at 800 rpm followed by 10 min at 1600 rpm, 4 °C, minicentrifuge HAWK 15/05). The upper plasma layers were carefully aspirated and to minimize oxidation processes immediately aliquoted (three portions per sample) to Eppendorf tubes kept at −20 °C in a cold box. Within 30 min, the plasma samples were transferred to −85 °C and stored at this temperature until analysis of isoprostanes.

### 3.4. Sample Preparation for Chromatographic Analyses

Aliquots of plasma (20–100 μL) spiked with 5 μL of methanolic solution of I.S. (8*-iso*-PGF_2α_-d_4_, 250 ng/mL) were diluted to 5 mL with deionized water and acidified to pH 3 with 1 M HCl (5 μL). A modified solid-phase extraction procedure based on [28] was used for the purification of isoprostanes from plasma samples. Briefly, Oasis HLB (10 mg) cartridge was pre-conditioned with 0.5 mL methanol, followed by 0.5 mL of 1 mM HCl. After loading a sample (5 mL), the cartridge was washed initially with 0.5 mL of 1 mM HCl, then with 0.5 mL of hexane. Isoprostanes along with I.S. were eluted with 1 mL of ethyl acetate containing 1% (*v*/*v*) of methanol. The eluate was evaporated under a stream of nitrogen to dryness. The residue was dissolved in 0.2 mL of a mixture containing acetonitrile/methanol/water (25:25:50).

### 3.5. HPLC-MS/MS Analysis

HPLC system (Agilent 1200 HPLC, Warsaw, Poland) directly coupled to a triple quadrupole mass spectrometer (API 4000, Applied Biosystems, Darmstadt, Germany). MS conditions were as described previously [29]. Instrument control data acquisition and data analysis were carried out with Analyst software (Applied Biosystem, Darmstadt, Germany). One hundred microliters of sample was injected onto a Zorbax Eclipse XDB column (100 mm × 4 mm; 3.5 μm). The mobile phase contained 0.01% (*v*/*v*) formic acid in ultrapure water (solvent A) and a mixture of methanol:acetonitrile 1:1 (solvent B). The gradient program for elution of isoprostanes was as follows: initial increase from 50% to 53% B over 0–4 min, then increased to 62% B over 1.5 min, further increase to 100% B over 0.5 min, 100% B for 6 min, finally the system was equilibrated for 8 min. Total time of analysis with equilibration of the system was 20 min. The column was maintained at 40 °C, the flow rate was 1 mL/min.

### 3.6. Statistical Analysis

Mean isoprostane concentrations and relative standard deviations (RSD) were calculated for 4 to 5 plasma samples analyzed in triplicate. When the volume of collected plasma was too small to perform reliable sample preparation, only 4 samples were used for calculations. A statistical program GraphPad PRISM 4 was used for data analysis. One-way ANOVA with Dunnett’s test was applied to determine the statistical significance of differences between samples derived from animals exposed to oxidative stress and control non-treated animals, while *t*-test to compare the effects of the two oxidative stress inducers investigated. For all comparisons, differences were considered significant at a value of *p* < 0.05.

## 4. Conclusions

In conclusion, the developed chromatographic LC–ESI-MS/MS protocol, being reasonably simple and quick, enables quantitative determination of four isoprostane regioisomers that are biomarkers of *in vivo* exposure to oxidative stress. All target compounds can be measured simultaneously in a multiple reaction monitoring mode (MRM) within a 20 min assay. MRM mode also reduces chemical noise, which leads to higher mass spectral sensitivity and selectivity. Another advantage of this protocol is that it requires only a small amount of biological material (in the range of 20–200 μL), which is very important in most experiments involving biological material. Our method can also be used for plasma samples containing analytes at a wide concentration level ranging from 0.05 up to 5 ng/mL (or higher with dilution step including). Compared to the GC/MS procedures widely used for the isoprostane determination, the present method involves fewer steps, because of the omission of derivatization and complicated sample purification. Consequently, it is less prone to sample losses and artifact generation. Moreover, in LC-MS and LC-MS/MS, most researchers have focused on only one main isoprostane that occurs in biological fluids [30,31]. In our study we propose a method for four isomers determination which can give a different response for oxidative stress. The additional non-identified isoprostane peaks (with the same mass transitions) detected on chromatograms and clearly separated from the target analytes, suggest that this method may also be used for the determination of other AA-derived isoprostanes, thereby broadening its applicability for lipidomic and/or metabolomic studies.

## Figures and Tables

**Table 1:** Calibration data of four isoprostanes determined using column Zorbax Eclipse XDB column (100 mm × 4 mm; 3.5 μm).

Analyte	Curve equation		*R^2^*	LOD (pg/mL)	LOQ (pg/mL)

	*Plasma sample*	*Water sample*			
8,15-isoP	*y* = 0.000084*x* + 0.0019	*y* = 0.000084*x* + 0.0006	0.999	15	50
8*-iso*P	*y* = 0.000094*x* + 0.015	*y* = 0.000094*x* + 0.011	0.999	12	40
11*-iso*P	*y* = 0.000075*x* + 0.00053	*y* = 0.000075*x* + 0.00041	0.999	17	51
15*-iso*P	*y* = 0.000070*x* + 0.0013	*y* = 0.000070*x* + 0.0008	0.999	15	50

**Table 2:** Recoveries (%) and relative standard deviations RSD (%) obtained by SPE-LC-MS/MS analysis of plasma samples fortified with a standard solutions of four isoprostanes at three spiking levels (50, 100 and 250 pg/mL).

Analyte	Recovery (%) †,‡		

	50 pg/mL	100 pg/mL	250 pg/mL
8,15*-iso*P	98.8 ± 5.4	99.5 ± 3.4	99.8 ± 1.2
8*-iso*P	100.6 ± 5.1	99.5 ± 3.2	100.7 ± 1.9
11*-iso*P	99.4 ± 7.8	97.9 ± 2.3	99.5 ± 2.7
15*-iso*P	99.2 ± 8.6	100.9 ± 2.5	99.4 ± 1.8

† Mean ± RSD.

‡ Each analysis was repeated three times (*n* = 3).

**Table 3:** Intra-day repeatability and inter-day reproducibility of assay, analyzed three times a day on three consecutive days.

Analyte	Recovery (%) †,‡			

	Intra-day			Inter-day
8,15*-iso*P	98.9 ± 3.8	98.2 ± 2.9	100.6 ± 2.0	99.2 ± 1.2
8*-iso*P	99.9 ± 4.3	99.6 ± 3.0	100.1 ± 3.2	99.9 ± 0.2
11*-iso*P	97.2 ± 7.1	97.9 ± 1.9	100.2 ± 3.7	98.5 ± 1.6
15*-iso*P	109.0 ± 1.5	108.0 ± 3.9	113.3 ± 6.1	110.1 ± 2.6

† Mean ± RSD.

‡ Each analysis was repeated three times (*n* = 3).
